# Recombinant Pyrin Domain Protein Attenuates Airway Inflammation and Alleviates Epithelial-Mesenchymal Transition by Inhibiting Crosstalk Between TGFβ1 and Notch1 Signaling in Chronic Asthmatic Mice

**DOI:** 10.3389/fphys.2020.559470

**Published:** 2020-10-23

**Authors:** Zhiguang Wang, Liangchang Li, Chongyang Wang, Yihua Piao, Jingzhi Jiang, Li Li, Guanghai Yan, Hongmei Piao

**Affiliations:** ^1^Jilin Key Laboratory for Immune and Targeting Research on Common Allergic Diseases, Yanbian University, Yanji, China; ^2^Department of Respiratory Medicine, Affiliated Hospital of Yanbian University, Yanji, China; ^3^Department of Anatomy, Histology and Embryology, Yanbian University Medical College, Yanji, China; ^4^Department of Intensive Care Unit, Affiliated Hospital of Yanbian University, Yanji, China

**Keywords:** recombinant pyrin domain protein, airway inflammation, epithelial-mesenchymal transition, airway remodeling, asthma

## Abstract

This article aims to investigate the effects of recombinant pyrin domain (RPYD) on airway inflammation and remodeling in mice with chronic asthma. The chronic asthma BALB/c mouse model was first sensitized by ovalbumin (OVA) and then challenged by OVA nebulization. RPYD or dexamethasone was given before OVA challenge. Our results showed that RPYD significantly inhibited the increase of total cell number, eosinophils, neutrophils and lymphocytes in bronchoalveolar lavage fluid (BALF) induced by OVA, and reduced the infiltration of inflammatory cells, the proliferation of goblet cells and collagen deposition. In addition, RPYD inhibited the mRNA and protein levels of α-smooth muscle actin (α-SMA), transforming growth factor (TGF)-β1, Jagged1, Notch1, Hes1 and Smad3, as well as Smad3 phosphorylation. TGFβ1 down-regulated the level of E-cadherin and promoted the expression of α-SMA, thus inducing epithelial-mesenchymal transition (EMT) in bronchial epithelial cells. We found that RPYD reduced EMT by inhibiting TGFβ1/smad3 and Jagged1/Notch1 signaling pathways. Further overexpression of NICD showed that under the stimulation of TGFβ1, NICD enhanced the phosphorylated Smad3 and nuclear Smad3, accompanied by the increased expression of Notch1 target gene Hes1. In contrast, after treatment with smad3 siRNA, the expression of Hes1 was down regulated as the decrease of Smad3, which indicates that there is crosstalk between smad3 and NICD on Hes1 expression. In conclusion, RPYD reduces airway inflammation, improves airway remodeling and reduces EMT in chronic asthmatic mice by inhibiting the crosstalk between TGFβ1/smad3 and Jagged1/Notch1 signaling pathways.

## Introduction

Bronchial asthma is a chronic airway inflammation that involves multiple cells (including eosinophils, macrophages, lymphocytes, neutrophils, etc.). The release of inflammatory factors by inflammatory cells promotes increased airway mucus secretion and goblet cell metaplasia (Li et al., 2019). In addition, damage to epithelial cells and the proliferation of bronchial smooth muscle cells may eventually develop into airway remodeling (Li et al., 2019). Epithelial-mesenchymal transition (EMT) is particularly important during the airway remodeling of asthma. The pathological manifestation of EMT is loss of epithelial cell phenotype and conversion to active mesothelial cells by downregulating epithelial markers E-cadherin and upregulating mesenchymal proteins α-SMA, ultimately leading to further impairment of airway barrier function and aggravating airway stenosis (Ijaz et al., 2014; Liu et al., 2017).

Transforming growth factor beta (TGF-β) is a cytokine involved in a variety of cellular events, including cell differentiation, migration, apoptosis, angiogenesis, and immunomodulation (Martelossi Cebinelli et al., 2016). There are three homologous isoforms of TGF-β (TGFβ-1, TGFβ-2, and TGFβ-3) and among them, TGFβ-1 is the most widely expressed isoform. In asthma models, abnormally expressed TGF-β1 induces epithelial cell damage, promotes neovascularization, and induces epithelial cell differentiation, ultimately promoting collagen deposition and the development of EMT (Qu et al., 2012; Ojiaku et al., 2017). The TGF signaling pathway is mediated through specific transmembrane type I and type II serine/threonine kinase receptors. Constitutively activated TGF type II receptors recruit and phosphorylate type I TGF receptors. TGFβ/Smad is closely related to EMT development. Phosphorylated receptor activated-Smad (including Smad2, Smad3) oligomerizes with Smad4 and is transferred into the nucleus to promote the expression of transcription factors (such as Snail2/Slug, Twist family, etc.), thus promoting the development of EMT (Kim et al., 2018; Tsubakihara and Moustakas, 2018).

Evolutionary conserved Notch signal pathways participate in cell growth, differentiation and apoptosis (Bray, 2016). There are four Notch receptors (Notch1–4) and five Notch ligands, including two ligands of the Jagged family (Jagged1 and Jagged2) and three delta-like ligands (Dll1, Dll3, and Dll4). Ligand-receptor interaction is a prerequisite for Notch signal activation (Bray, 2016). After binding and activation, the intracellular part of Notch receptor is digested by γ-secretase to release the soluble Notch intracellular domain (NICD). NICD can enter the nucleus and combine with the DNA-binding protein RBP-Jκ (also known as CSL) to form a complex that initiates the transcription process of target genes such as Hes1 (LaFoya et al., 2016). Notch1 is involved in the development of EMT in tumor and lung fibrosis models (Pal et al., 2018; Yin et al., 2018). In addition, TGF-β and Notch pathways are synergistically involved in regulating Hes1 expression (Blokzijl et al., 2003). Moreover, TGF-β1 and Notch1 are also involved in the development of EMT in epithelial ovarian cancer cells (Zhou et al., 2016). In human primary keratinocytes, TGF-β1 and Notch1 synergistically regulate the expression of the receptor protein tyrosine phosphatase-κ gene (Xu et al., 2015). However, whether TGF-β1 and Notch1 have a synergistic effect in the occurrence of EMT in chronic asthma is less reported.

The pyrin domain (PYD) belongs to the death domain fold protein superfamily. PYD act as a homotypic protein-protein interaction domain and is involved in inflammasome assembly (Dorfleutner et al., 2015). The Nod-like receptor NACHT, LRR, and PYD domain-containing protein 3 (NLRP3) inflammasome, as a cytoplasmic signal complex, participates in a variety of inflammatory diseases, mediates the activation of inflammatory reactions, and is required for the activation of allergen-specific Th2 cells in allergic asthma (Bruchard et al., 2015). Inflammasome assembly requires the adaptor ASC that contains PYD and depends on the interaction between PYD-PYD. Interestingly, many studies have now demonstrated that POP2 (PYRIN domain only protein 2) blocks the recruitment of ASC to upstream sensors by binding to ASC, thereby preventing caspase-1 activation and cytokine release, blocking the NF-κB signaling pathway, and inhibiting inflammasome assembly (Periasamy et al., 2017; Ratsimandresy et al., 2017). Thus, we speculate that exogenous administration of “pyrin-only” proteins might affect PYD-protein interactions, which in turn modulate the NF-κB signaling pathway and alleviate the inflammatory response. This is confirmed by our previous study that recombinant pyrin domain protein (RPYD) attenuated OVA-induced inflammatory response in asthmatic mice by inhibiting NF-κB signaling (Piao et al., 2019). It is recently reported that knockdown of NLRP3 in human renal tubular cells alleviated TGFβ1-induced EMT (Song et al., 2018). This finding has inspired us to verify whether RPYD could affect the occurrence of EMT in airway remolding asthma mice.

Therefore, in this study, we focused on the role of RPYD in airway inflammation and EMT of airway remodeling in asthmatic mice. The underlying mechanism involving TGF-β1 and Notch1 pathways was further analyzed and discussed.

## Materials and Methods

### Mice

Male BALB/c mice (aged 8–10 weeks, weight 20 ± 2 g) were obtained from the Animal Experiment Center of Yanbian University (Yanji, China). The experimental procedures were performed according to the Regulations on the Administration of Laboratory Animals and approved by the Ethics Committee of our University.

### Asthma Model Establishment, Drug Administration, and Animal Grouping

**FIGURE 1:**
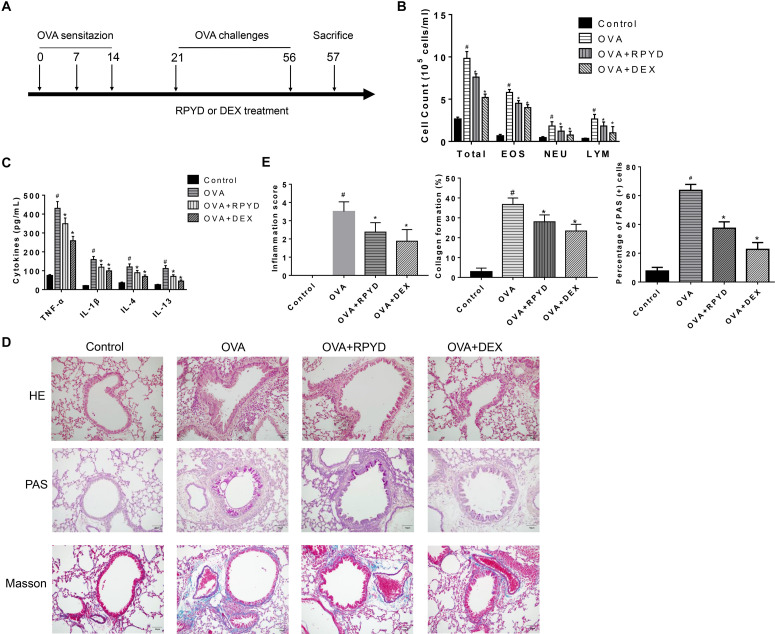
Effects of RPYD on OVA-induced inflammation of asthmatic mice. **(A)** Schematic figure for *in vivo* experiments. **(B)** The numbers of total cells and cellular components in bronchoalveolar lavage fluids (BALFs) was counted. EOS, eosinophils; NEU, neutrophils; LYM, lymphocytes. **(C)** The expression levels of inflammatory cytokines TNF-α, IL-1β, IL-4, and IL-13 in BALF were measured by ELISA. Data were shown as mean ± *SD* (*n* = 8). ^#^*P* < 0.05 vs. Control mice, ^∗^*P* < 0.05 vs. OVA mice. **(D)** Lung tissue sections were stained with hematoxylin-eosin (HE) to observe inflammatory cell infiltration and scored for inflammation. Periodic acid-Schiff (PAS) was used to assess goblet cell hyperplasia. Subepithelial collagen deposition and fibrosis were assessed by Masson staining. The magnification was 200×. Scale bar: 50 μm. **(E)** Inflammation score, collagen deposition and percentage of PAS (+) cells.^ #^*P* < 0.05 vs. Control mice, ^∗^*P* < 0.05 vs. OVA mice.

Thirty-two male BALB/c mice were randomly selected and divided into four groups, including control group, Ovalbumin (OVA) group, OVA + RPYD protein treatment group (OVA + RPYD), and OVA + dexamethasone treatment group (OVA + DEX), with 8 mice in each group. The time line for model establishment and drug administration was shown in Figure 1A. Briefly, each mouse in the control group was intraperitoneally injected with 200 μL saline on days 1, 7, and 14. Mice in the remaining three groups were sensitized with 200 μL of sensitization solution (OVA 10 μg + AlOH 1 mg) by intraperitoneal injection on days 1, 7, and 14. From day 21, mice were placed in a transparent nebulization device and challenged with 3% OVA nebulization for 30 min three times a week until Day 56. Mice in control group were challenged with the same amount of normal saline instead. The OVA+RPYD group received intranasal RPYD protein (200 μg/kg), which was prepared and purified as previously described (Piao et al., 2019), 1 h before challenge, and the OVA+DEX group received intraperitoneal dexamethasone (1 mg/kg). Mice in the control group and OVA group received equal volume of saline. Mice were euthanized 24 h after the last challenge. Bronchoalveolar lavage fluid (BALF) and lung tissues were taken for further analysis.

### Determination of Cell Count and Inflammatory Factors in BALF

The lungs were lavaged with 0.8 mL PBS to collect BALF. The total cell number in 0.05 mL aliquots of BALF was counted using a hemocytometer (Baxter Diagnostics, Deerfield, IL, United States). The remaining samples were centrifuged and the supernatants were collected and stored at −80°C until use. BALF cells were smeared after resuspension of the cell pellet in PBS, stained with Diff-Quik’s solution (International Reagents, Kobe, Japan) and sorted by cell morphology and staining characteristics. The levels of tumor necrosis factor alpha (TNF-α), IL-1β, IL-4, IL-13 in BALF were determined using mouse ELISA kits (R&D Systems, Minneapolis, MN, United States) according to the manufacturer’s instructions.

### Histological Staining

The left lung tissue was embedded in paraffin and cut into 5 μm sections. Hematoxylin-eosin staining was performed according to routine experimental procedures for analysis of histomorphological changes and scoring of inflammation. The severity of peribronchial inflammation was graded semi-quantitatively using the following criteria: 0 point, normal; 1 point, few cells; 2 points, a ring of inflammatory cells of 1 cell layer deep; 3 points, a ring of inflammatory cells of 2–4 cells deep; 4 points, a ring of inflammatory cells of 4 cells deep. Periodic acid-Schiff (PAS) staining was also performed to observe goblet cells proliferation and mucus production. The number of PAS-positive cells (goblet cells) and the number of epithelial cells were counted. According to previous studies (Fulkerson et al., 2006; Wang et al., 2018), the percentage of PAS-positive goblet cells was calculated as the ratio of the number of goblet cells to the total number of epithelial cells in the bronchus. Collagen deposition around the airways was observed by Masson’s trichrome staining and quantified as previously described (Cho et al., 2004a, b). Airway basement membrane perimeter was measured using image-Pro Plus 6.0, and the area of trichrome staining around the bronchi in the lungs was delineated and quantified. The percentage of collagen deposition was expressed as the area of trichrome staining per micron length of basement membrane of bronchioles 150–200 μm of internal diameter. Quantitative analysis for hematoxylin-eosin staining, PAS staining and Masson’s trichrome staining were performed in 5 randomly selected fields at 200 × magnification.

### Immunohistochemistry

Lung tissue sections were incubated with primary antibodies against α-smooth muscle action (α-SMA) (1:320 dilution, CST, 19245, United States), TGF-β1 (1:100 dilution, Abcam, ab92486, Cambridge, MA, United States), Notch1 (1:400 dilution, CST, 3608, United States), followed by peroxidase conjugated secondary antibody (goat anti-rabbit IgG-HRP, 1:500 dilution, Abcam, ab205718). After immunostaining, sections were counterstained with hematoxylin and mounted. Five fields were randomly selected at 200 × magnification and photographed (Eclipse E600, Nikon, Japan). Airwayα-SMA expression was evaluated using Image-Pro Plus 6.0 analysis software as previously described (Li et al., 2020). Qualification of α-SMA was accomplished by the area of α-SMA immunostaining per um length of the basement membrane of bronchioles (μm^2^/μm).

### RT-PCR

Total cellular RNA was isolated from lung tissue using TRIzol reagent (Invitrogen, Carlsbad, CA, United States). Complementary DNA was reverse transcribed by Oligo (dT) 15 primer and AMV reverse transcriptase (Takara, Otsu, Shiga, Japan). The primer sequences for RT-PCR were listed in Table 1. The reaction conditions were as follows: 95°C for 2 min, followed by 40 cycles of 95°C for 10 s, 60°C for 1 min, and 72°C for 30 s. RT-PCR results were quantified using the threshold cycle (Ct) method. The relative expression of the target gene was calculated using 2^–△△^^*CT*^ method. GAPDH was used as internal control.

**TABLE 1:** RT-PCR primers.

Genes	Primers	Sequence (5′–3′)
TGFβ1	Forward	CCTTGCCCTCTACAACCAACAC
	Reverse	CTTGCAGGAGCGCACGATC
Smad3	Forward	ATGTCAACAGGAATGCAGCAGTGG
	Reverse	ATAGCGCTGGTTACAGTTGGGAGA
Notch1	Forward	CGCACAAGGTGTCTTCCAG
	Reverse	CGGCGTGTGAGTTGATGA
NICD	Forward	TGAGACAGGCAACAGTGAAG
	Reverse	CAGCATCTGAACGAGAGTATCG
Hes1	Forward	TTGGAGGCTTCCAGGTGGTA
	Reverse	GCCCCGTTGGGAATGAG
α-SMA	Forward	GGCTGTTTTCCCATCCATTGT
	Reverse	TCTTTTGCTCTGTGCTTCGT
E-cadherin	Forward	CGTAGCAGTGACGAATGTGGTAC
	Reverse	AACTGGAGAACCATTGTCTGTAGC
GAPDH	Forward	CAAGGTCATCCATGACAACTTTG
	Reverse	GTCCACCACCCTGTTGCTGTAG

### Cell Culture and Treatment

The human bronchial epithelial cell line BEAS-2B was purchased from the American Type Culture Collection (Rockville, MD, United States). They were cultured in DMEM containing 10% fetal bovine serum (Gibco BRL) and 1% penicillin/streptomycin (Gibco BRL) at 37°C with 5% CO_2_. They were stimulated with recombinant human TGF-β1 (Abcam, ab166886) at 10 ng/mL for 48 h to induce EMT as previously described (Itoigawa et al., 2015; Haddad et al., 2019). During some experiments, BEAS−2B was pretreated with RPYD (5 μmol/L), TGFβ1 inhibitor SB431542 (10 μM, CST, #14775, United States), or NOTCH1 inhibitor DAPT (10 μM, CST, #15020, United States) for 6 h before TGF-β1 stimulation.

### Smad3 siRNA and NICD Overexpression

For NICD overexpression, a lentivirus (LV) plasmid containing NICD was constructed by Genechem Company (Shanghai, China) and named “LV-NICD.” Meanwhile, the “LV-Control” was used as control. BEAS-2B cells were transfected with LV plasmids for 72 h. For silencing of Smad3 expression, cells were co-transfected with 1.5 μM of Smad3 siRNA (Santa Cruz Biotechnology, SC-38376) or negative control siRNA (Santa Cruz Biotechnology, sc-37007) for 48 h. The transfection efficiency was verified by RT-PCR and Western blot.

### Western Blot

Total protein was extracted from lung tissues and cells. Cytoplasmic and nuclear proteins were extracted from cells as previously described (Li et al., 2015). Protein concentration was determined and quantified by Bradford reagent (Bio-Rad, Hercules, CA, United States). Then proteins were separated by SDS-PAGE and transferred to PVDF membranes. After incubation with 5% skim milk at room temperature, the membranes were incubated with the corresponding primary antibodies overnight at 4°C, followed by incubation with horseradish peroxidase-conjugated secondary antibodies at room temperature for 2 h. ECL (Beyotime, Beijing, China) was used for color development. Protein bands were analyzed using a Gel Doc XR system (Bio-Rad, CA, United States). The antibodies of phosphor-Smad3 (#9520), Smad3 (#9523), Jagged1 (#70109), Notch1 (#3608), NICD (#4147), Hes1 (#11988), CSL (#5313), α-SMA (#19245), E-cadherin (#3195), GAPDH (#5174), and PARP (#9532) were all purchased from Cell Signaling Technology (Beverly, MA, United States). The anti-TGFβ1 (ab92486) and goat anti rabbit secondary antibody (ab205718) were purchased from Abcam (Cambridge, MA, United States).

## Statistical Analysis

Data were expressed as mean ± *SD* and analyzed using SPSS 22.0. Comparisons between two groups were performed using the independent *t*-test, and comparisons among multiple groups were conducted using ANOVA followed by Dunnett’s *post-hoc* test. *P* < 0.05 was considered statistically significant. GraphPad Prism 7.0 was used to plot graphs.

## Results

### RPYD Improves OVA-Induced Inflammation and Reduces Inflammatory Factor Levels in BALF of Asthmatic Mice

Asthma is a chronic airway inflammation that is mainly characterized by the infiltration of inflammatory cells in the lung airways and subepithelial space. Thus, we used OVA to establish a mouse model of chronic asthma and studied the effect of RPYD on airway inflammation. Compared with control, the number of total inflammatory cells, eosinophils, neutrophils, and lymphocytes in BALF after OVA challenge was significantly higher (Figure 1B). ELISA results showed that TNF-α, IL-1β, IL-4 and IL-13 were highly expressed in BALF after OVA challenge. However, after RPYD and DEX treatment, the total cell count, eosinophil count, neutrophil count and lymphocyte number as well as the inflammatory factors of TNF-α, IL-1β, IL-4, and IL-13 in BALF decreased significantly (Figure 1C). The above results indicate that RPYD reduces the inflammatory response of the respiratory tract.

Further assessment of histological changes in the lungs was performed (Figures 1D,E). As shown by HE staining, compared with control, there were extensive peri-bronchial and vascular infiltration of inflammatory cells, and the inflammation score was significantly increased after OVA challenge. However, RPYD and DEX treatment reduced the infiltration of inflammatory cells and inflammation score, suggesting that RPYD reduces airway inflammation caused by OVA. PAS staining further confirmed the effect of RPYD on mucus secretion. The number of PAS-stained epithelial goblet cells in OVA-induced mice was significantly higher than that in the control mice, and both RPYD and DEX could effectively reduce the number of PAS-stained goblet cells. The effect of RPYD on collagen deposition was also assessed by Masson’s trichrome staining. The results showed that collagen accumulation induced by OVA in the airway and vascular stroma of mice was obviously increased, and RPYD and DEX treatment effectively reduced the airway collagen deposition. The above results indicate that RPYD could reduce inflammatory cytokines, airway inflammatory cell infiltration, goblet cell proliferation, and collagen deposition of chronic asthmatic mice induced by OVA.

### RPYD Reduces Expression of EMT-Related Proteins and Inhibits Expression Proteins in TGFβ1 and NOTCH1 Pathways Induced by OVA

**FIGURE 2:**
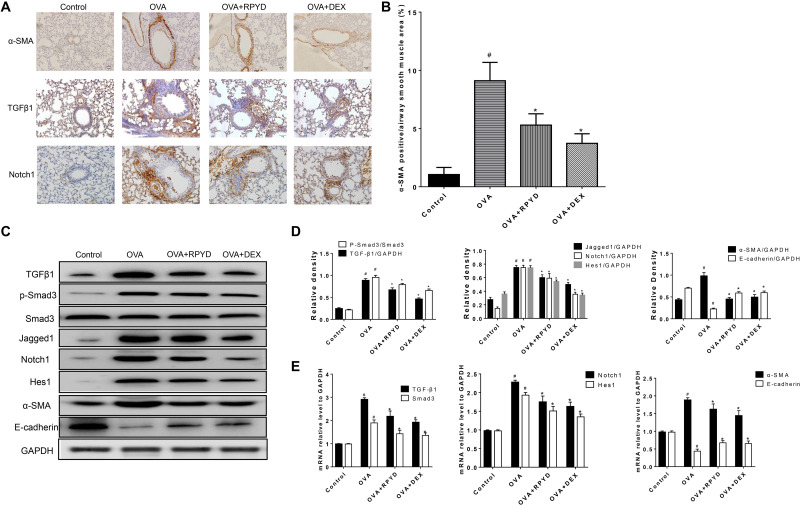
Effects of RPYD on α-SMA, TGF-β and Notch1 expression in lung tissue. **(A)** Immunohistochemical staining was used to assess the distribution of α-SMA, TGFβ1, Notch1 expression in lung tissue. The magnification was 200×. Scale bar: 50 μm. **(B)** Quantitative analysis of α-SMA positive area in airway smooth muscle. **(C)** Western blot analysis of TGFβ, Smad3, phospho-Smad3, Jagged1, Notch1, Hes1, α-SMA, E-cadherin protein levels with GAPDH as a standard control. **(D)** Results of band gray scale analysis of these proteins. **(E)** Quantitative RT-PCR was performed to determine the mRNA levels of TGFβ, Smad3, Notch1, Hes1, α-SMA, E-cadherin in lung tissue. Data are shown as mean ± *SD* (*n* = 8). ^#^*P* < 0.05 vs. control mice, *P < 0.05 vs. OVA mice.

Airway narrowing caused by airway remodeling leads to irreversible airway obstruction, of which EMT plays an important role (Liu et al., 2017). Therefore, the role of RPYD in EMT of airway remodeling is further elucidated. By immunohistochemical staining (Figures 2A,B), western blot (Figures 2C,D) and RT-PCR (Figure 2E), we analyzed the levels of a-SMA and E-cadherin. OVA challenge caused a significant increase in a-SMA staining around the airways. In addition, through Western blot analysis and RT-PCR, we further confirmed the up-regulation of a-SMA and the down-regulation of E-cadherin in lung tissue, all of which indicated that OVA induced EMT in mice. After treatment with RPYD, the expression of a-SMA was significantly reduced whereas the expression of E-cadherin was significantly up-regulated, suggesting that the progression of OVA-induced EMT is reduced by RPYD.

The role of TGFβ1 and Notch1 in EMT has been confirmed in numerous studies (Chen et al., 2017; Jiang et al., 2019). Therefore, we investigated whether the inhibitory effect of RPYD on airway remodeling is related to its regulation of TGF-β1 and Notch1 levels. Western blot and RT-PCR results showed that after OVA challenge, the levels of TGFβ1 and Notch1 were significantly increased. At the same time, the phosphorylation of Smad3, which is downstream of TGFβ1, was significantly increased. In addition, Jagged1, which is a promoter of Notch1, and Hes1, which is a target gene of Notch1, were also significantly increased. However, RPYD treatment significantly reduced the levels of these proteins, suggesting that RPYD is involved in inhibiting airway remodeling by regulating the TGFβ1 and Notch1 signaling pathways.

### RPYD Reduces the EMT by Inhibiting the Phosphorylation of Smad3 and Its Nuclear Translocation

**FIGURE 3:**
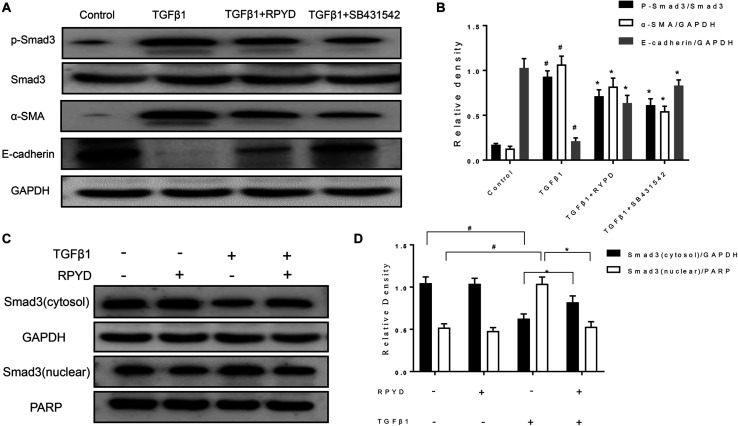
Effects of RPYD on TGFβ1/Smad3 signaling in bronchial epithelial BEAS-2B cells induced by TGFβ1. BEAS-2B cells were treated as described in section “Materials and Methods.” **(A)** Western blot was used to detect the protein expression of phosphorylated Smad3, Smad3, α-SMA and E-cadherin. **(B)** Relative level of each protein. All data were shown as mean ± *SD* (*n* = 3). ^#^*P* < 0.05 vs. Control group, *P < 0.05 vs. TGFβ1 stimulation group. **(C)** Western blot results of cytosol and nuclear expression of Smad3. **(D)** Relative level of cytosol and nuclear Smad3.

Studies have shown that TGFβ1 induces and promotes the occurrence of EMT (Haddad et al., 2019; Song et al., 2018). Therefore, TGFβ1 was used as an agonist to induce the occurrence of EMT in BEAS-2B cells. As shown in Figures 3A,B, TGFβ1 induced an increase in α-SMA and reduced the level of E-cadherin in BEAS-2B cells. RPYD significantly reversed these changes. To further reveal the mechanism of RPYD on TGF-β1-induced EMT, we examined the phosphorylation level of Smad3 and found that Smad3 phosphorylation increased after TGFβ1 stimulation, while RPYD inhibited Smad3 phosphorylation. Similar results were obtained in the group using the TGFβ1 inhibitor SB431542. Furthermore, RPYD treatment alone did not significantly affect the cytosol and nuclear levels of Smad3 (Figures 3C,D). However, TGF-β1 treatment alone significantly decreased the cytosol levels of Smad3 while increased nuclear levels of Smad3. In cells treated with TGF-β1 and RPYD, the level of Smad3 in the nucleus was reduced whereas in cytosol was increased. This data indicates that RPYD may inhibit the occurrence of EMT by regulating the TGFβ1/Smad3 signaling pathway.

### RPYD Reduces EMT by Inhibiting the Jagged1/Notch1 Signaling Pathway

**FIGURE 4:**
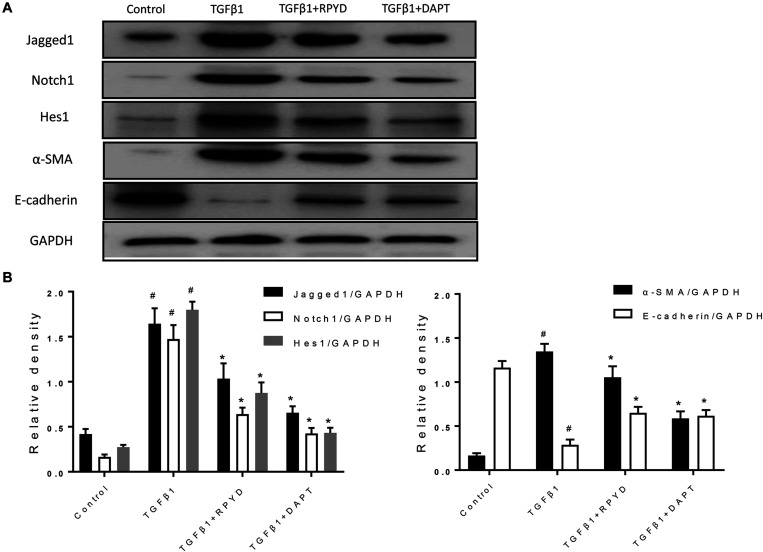
Effects of RPYD on Jagged1/Notch1 signaling in bronchial epithelial BEAS-2B cells induced by TGFβ1. BEAS-2B cells were treated as described in section “Materials and Methods.” **(A)** Western blot was used to detect the protein expression of Jagged1, Notch1, Hes1, α-SMA and E-cadherin. **(B)** Relative level of each protein. All data were shown as mean ± *SD* (*n* = 3). ^#^*P* < 0.05 vs. Control group, *P < 0.05 vs. TGFβ1 stimulation group.

As previously mentioned, Notch1 also plays a critical role in the development of EMT (Pal et al., 2018). The Jagged1/Notch1 signaling pathway was therefore also explored. In Figure 4, after induction by TGFβ1, the expression of α-SMA was up-regulated and conversely, E-cadherin expression was decreased, indicating the occurrence of EMT. The levels of Jagged1, Notch1 and Hes1 proteins were significantly increased after induction by TGFβ1, suggesting that Jagged1/Notch1 pathway is involved in the occurrence of EMT. Conversely, treatment with RPYD and DAPT, as a Notch1 inhibitor, significantly inhibited these proteins and reduced EMT. The above results indicate that RPYD might inhibit the occurrence of EMT by regulating the Jagged1/Notch1 signaling pathway.

### NICD Overexpression Promotes the Phosphorylation and Nuclear Translocation of Smad3

**FIGURE 5:**
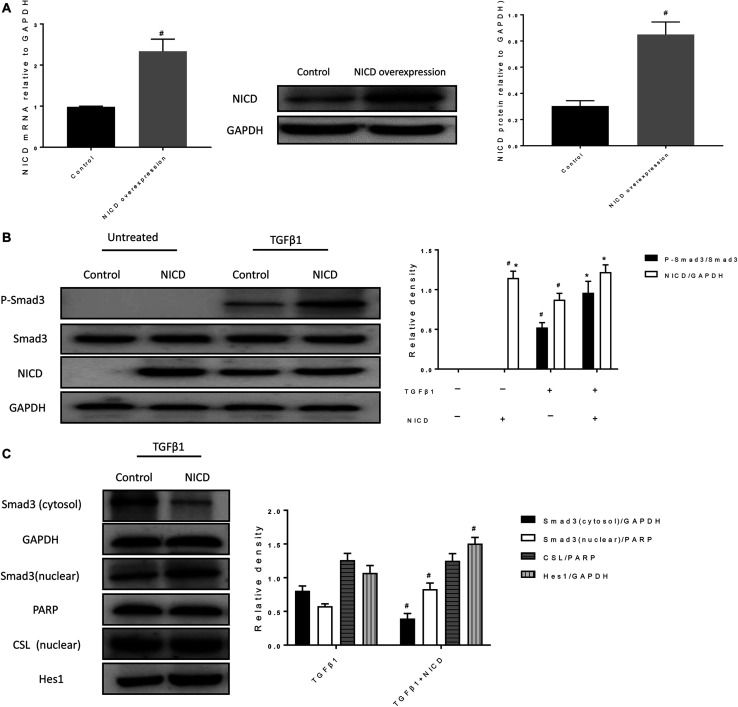
NICD overexpression promotes the phosphorylation and nuclear translocation of Smad3. **(A)** Expression of NICD in transfected cells was verified by RT-PCR and western blotting after BEAS-2B cells were infected with lentivirus for 24 h. All data were shown as mean ± *SD* (*n* = 3). ^#^*P* < 0.05 vs. Control group. **(B)** Western blot was used to detect phospho-Smad3, Smad3, and NICD protein expression. The band intensity of the protein was expressed as a ratio to GAPDH. All data were shown as mean ± *SD* (*n* = 3). ^#^*P* < 0.05 vs. Control group, *P < 0.05 vs. TGFβ1 stimulation group. **(C)** Western blot was used to detect Smad3, CSL, and Hes1 protein expression. The band intensity was expressed as a ratio to GAPDH or PARP. All data were shown as mean ± *SD* (*n* = 3). ^#^*P* < 0.05 vs. TGFβ1 stimulation group.

Previous studies have confirmed that TGFβ1 pathway interacts with Notch1 pathway (Tang et al., 2010; Shang et al., 2016). Thus, we further investigated whether these is such interaction in BEAS-2B cells during EMT. In Figure 5A, a cell line overexpressing NICD was successfully constructed by lentiviral transfection. As shown in Figure 5B, the phosphorylation of Smad3 was hardly seen in cells without TGFβ1 stimulation, and overexpression of NICD did not significantly alter the phosphorylation level of Smad3. In TGFβ1-stimulated cells, Smad3 phosphorylation was significantly increased compared to un-stimulated cells, whereas overexpression of NICD further enhanced Smad3 phosphorylation but did not significantly alter total Smad3 levels. Next, we observed that overexpression of NICD resulted in increased nuclear expression of Smad3 after TGFβ1 stimulation, accompanied by increased Hes1 expression (Figure 5C). There was no significant difference in nuclear CSL levels. These results show that NICD, as an activated intracellular fragment of Notch1, enhances intracellular phosphorylation of Smad3 and nuclear expression of Smad3.

### Smad3 Is Involved in the Expression of the Notch1 Target Gene Hes1

**FIGURE 6:**
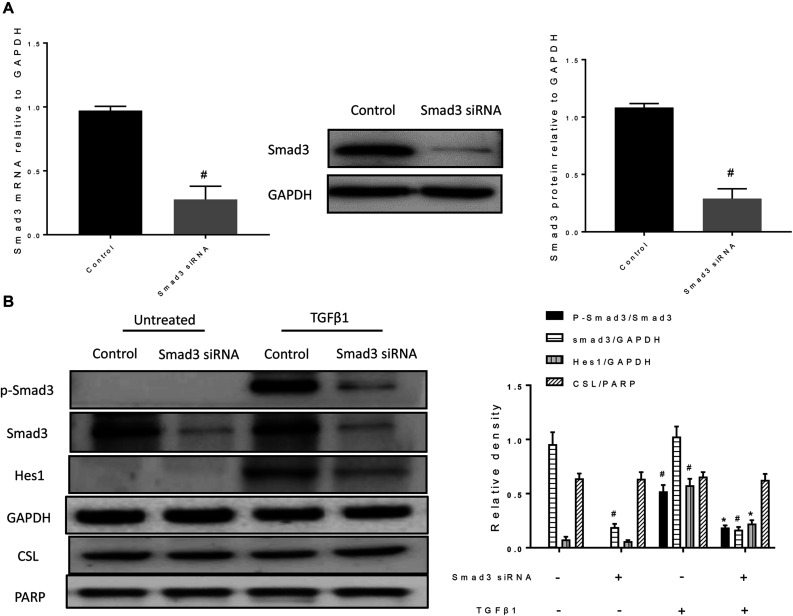
Smad3 siRNA reduces the level of Smad3 phosphorylation and decreases the protein expression of Hes1. **(A)** The specific knockdown of Smad3 by siRNA was verified by RT-PCR and western blotting. All data were shown as mean ± *SD* (*n* = 3). ^#^*P* < 0.05 vs. Control group. **(B)** Western blot was used to detect phospho-Smad3, Smad3, Hes1, and CSL protein expression. The band intensity was expressed as a ratio to GAPDH or PARP. All data were shown as mean ± *SD* (*n* = 3). ^#^*P* < 0.05 vs. Control group, *P < 0.05 vs. TGFβ1 stimulation group.

Next, we examined whether loss of smad3 affects CSL and Hes1 expression. After treatment with Smad3 siRNA, both mRNA and protein expression levels of Smad3 were significantly reduced (Figure 6A). In the TGFβ1 + Smad3 siRNA group, no significant phosphorylation of Smad3 was observed after stimulation with TGFβ1. However, Hes1 was also decreased (Figure 6B). However, nuclear CSL levels were unchanged by either TGFβ1 stimulation or Smad3 siRNA treatment. The above results indicate that Smad3 might cooperate with NICD to induce the expression of Hes1 and synergically promote the occurrence of EMT.

## Discussion

In this study, we explored the effects of RPYD on chronic airway inflammation and airway remodeling in chronic asthma. We found that RPYD significantly reduced the number of inflammatory cells and the release of inflammatory factors in BALF and reduced the inflammatory response around the airways. In airway remodeling, RPYD suppressed airway remodeling by reversing EMT, which may be achieved by inhibiting the TGFβ1/Smad3 and Jagged1/Notch1 signaling pathways. Meanwhile, there was a synergistic interaction between Smad3 and NICD on the expression of Hes1 during EMT. RPYD may reduce EMT by inhibiting this interaction.

The cytokines of IL-4, IL-5, and IL-13 produced by Th2 cells are deeply involved in IgE synthesis, eosinophil activation, mucus secretion, and airway remodeling during the pathogenesis of asthma (Manka and Wechsler, 2018; Ramsahai et al., 2019). Thus, IL-4 and IL-13 are factors that aggravate the inflammatory response in asthma, and there are also many studies that have achieved control of Th2-type asthma by preparing IL-4 and IL-13 antibodies (Bagnasco et al., 2016; Godar et al., 2018). In this study, IL-4 and IL-13 were also found to be increased in OVA asthma model, and RPYD could reduce the expression of IL-4 and IL-13 and thus inhibit airway inflammation. The infiltration of inflammatory cells around the airways and the release of inflammatory factors are the main factors in the pathogenesis of asthma (Mims, 2015). Eosinophil accumulation at the bronchial level, and release of cytotoxic granule proteins exacerbate the local inflammatory response and induce epithelial cell injury and collagen deposition (Matucci et al., 2018). Eosinophils contribute to tissue destruction, promote the progression of airway inflammation, and are also a major source of TGF-β (Fehrenbach et al., 2017). Pro-inflammatory cytokines (including TNF-α and IL-1β) also participate in aggravating the allergic response (Fahy, 2015). Thus, studies are now mainly focused on how to effectively control the production of inflammatory cells and inhibit the release of inflammatory factors (Fajt and Wenzel, 2015; Barnes, 2018). In this study, we induced chronic asthma in mice by OVA and investigated the therapeutic effect of RPYD on airway inflammation. It was found that RPYD could effectively reduce the number of inflammatory cells in BALF and inhibit the expression of inflammatory factors, thereby improving airway inflammation.

Airway remodeling in allergic bronchial asthma is considered to be a negative consequence of the chronic inflammatory response. Chronic inflammation leads to permanent airway barrier disruption, malformation and stenosis. Thus, chronic airway inflammation is considered as the main factor leading to airway remodeling (Hirota and Martin, 2013). Airway remodeling is mainly manifested as subepithelial cell collagen deposition, bronchial stenosis caused by goblet cell hyperplasia and hypertrophy, airway smooth muscle hypertrophy and proliferation, airway mucous gland proliferation; meanwhile, EMT is essential in airway remodeling caused by epithelial injury (Boulet, 2018). Here, in this study, we focused on the role of RPYD in EMT. In OVA-induced asthmatic mice, the increase in the EMT-related α-SMA and decrease in the epithelial cell marker E-cadherin were observed, and this was further reduced by RPYD. These results indicate that EMT is involved in airway remodeling, and RPYD could effectively reduce the EMT. By using *in vitro* experiments, it was also confirmed that RPYD could inhibit the EMT induced by TGFβ1.

Smad3 has also been found to promote the development of EMT by promoting the expression of transcription factors Snail2/Slug, Twist, etc. (Kim et al., 2018; Tsubakihara and Moustakas, 2018). However, the role of Smad3 in Hes1 expression is unclear. In the present study, we therefore focused on changes in Smad3 (Kou et al., 2017; Tsubakihara and Moustakas, 2018). We verified that RPYD inhibited the phosphorylation of Smad3 induced by TGFβ1 *in vitro*, and suppressed the expression of Smad3 in the nucleus. In addition, we found that SMAD3 knockdown by siRNA reduced the protein expression of Hes1, indicating that Smad3 participates in the signaling pathway events of NOTCH1, cooperatively regulates the expression of HES1, and promotes the occurrence of EMT.

Hes1, as a target gene of NICD, has also been confirmed to induce the occurrence of EMT (Sun et al., 2017; Liu et al., 2018). Therefore, we validated the role of RPYD in the NOTCH1 signaling pathway and found that RPYD inhibited NOTCH1 during reversal of EMT. In order to clarify the relationship between Smad3 and NICD, NICD was over expressed. The results showed that NICD itself did not cause the phosphorylation of Smad3, while in the presence of TGFβ1 stimulation, NICD could lead to the increase of Smad3 phosphorylation and increase of Smad3 nuclear expression, which was further accompanied by the increase of Hes1 expression and EMT marker α-SMA. However, the increase of Hes1 expression may also be caused by the overexpression of NICD, so the subsequent study verified that Smad3 also had a synergistic effect in Hes1 expression by Smad3 siRNA. Previous study has shown that Smad3 relies on the interaction with NICD during transcription of Hes1, which in turn recruits to CSL DNA binding sites and is involved in transcription of Notch target genes, illustrating that there is indeed a signaling crossover process between Smad3 and NICD (Xu et al., 2015).

In summary, RPYD attenuates airway inflammation and airway remodeling in asthmatic mice, possibly through inhibiting the interaction between crosstalk between TGFβ1/Smad3 and Jagged1/Notch1 signaling pathways. Our findings may provide evidence for the use of RPYD in bronchial asthma treatment.

## Data Availability Statement

The raw data supporting the conclusions of this article will be made available by the authors, ithout undue reservation.

## Ethics Statement

The animal study was reviewed and approved by the Ethics Committee of Yanbian University.

## Author Contributions

ZW, LL, CW, YP, JJ, and LL performed the study. ZW and LL analyzed and interpreted the data and wrote the manuscript. GY and HP designed the study and revised the manuscript. GY collected the funds. All authors contributed to the article and approved the submitted version.

## Conflict of Interest

The authors declare that the research was conducted in the absence of any commercial or financial relationships that could be construed as a potential conflict of interest.
